# Comprehensive clinical analysis of patients with primary malignant tumor of pituitary gland: A population-based study

**DOI:** 10.3389/fsurg.2022.933168

**Published:** 2022-10-18

**Authors:** Xu Sun, Lanqing Huo, Xin Wang, Chunlan Zhang, Ailin Zhao

**Affiliations:** ^1^ Department of Hematology, West China Hospital, Sichuan University, Chengdu, China; ^2^ Department of Radiation Oncology, Sun Yat-sen University Cancer Center, State Key Laboratory of Oncology in South China, Guangzhou, China

**Keywords:** primary malignant pituitary tumor, incidence, death, treatment, survival

## Abstract

**Background and Objectives:**

This study aims to perform a comprehensive clinical analysis of patients with primary malignant pituitary tumors (PMPT) that involves incidence, demographics, treatments, long-term survival, and death causes.

**Materials and methods:**

Patients with PMPT were identified from registries of the Surveillance, Epidemiology, and End Results (SEER) database. Frequencies and average annual age-adjusted rate (AAR) were calculated for incidence trend analyses using Join-point regression. Univariate and multivariate Cox regression analyses were conducted to identify potential prognostic factors associated with patients' survival outcomes. Using the Kaplan–Meier method and log-rank test, survival curves were plotted and compared, respectively. Propensity score matching (PSM) was performed to balance baseline characteristics.

**Results:**

The AAR for PMPT was 0.233 (95%CI: 0.205–0.264) per 1,000,000 using nine SEER registries from 1975 to 2017. The incidence trend has declined over years but without significance (–1.04% per year, *P* = 0.10). Besides, older age may indicate a higher incidence rate for both pediatric and adult patients. From 18 SEER registries, a total of 501 PMPT patients were also identified. Univariate and multivariate Cox regression showed age, sex, tumor extent, and marital status were independent prognostic factors for malignant pituitary tumors. Via PSM, we found that patients who received surgery, radiotherapy, and chemotherapy did not demonstrate significantly different survival than those who did not.

**Conclusion:**

This study first conducts a comprehensive clinical analysis of patients with PMPT and provides guide effects on future study designs. More studies should be conducted to focus on its characteristics and therapy.

## Introduction

Pituitary tumors were the second malignant disease that accounted for 10%–15% of intracranial neoplasms. With the wide application of radiological techniques such as computing tomography (CT) and magnetic resonance imaging (MRI), pituitary adenomas found by accident has been increasing. It was previously reported to have an estimated overall prevalence of 16.7% (1). The most common tumor located in the pituitary is adenoma, which can be often classified according to the size, invasion, differentiation, and endocrinal nature of the tumor in clinical practice. Most adenomas showed no invasive behavior, while other malignant types including adenocarcinoma, germinoma, chordoma, etc. occurred very rarely. Previously, very few studies focused on the primary malignant tumors located in the pituitary gland, which may demonstrate different clinical characteristics and survival outcomes.

According to the functional status, pituitary tumors can be divided into functioning and non-functioning. Among functioning cases, there can be a series of clinical symptoms such as acromegaly, Cushing disease, etc. For them, surgical resection is recommended as a first-line treatment (2–4). However, the space-occupying effect may play a more critical role in the management of non-functional tumors for the lack of endocrine functions. Thus, fewer patients need clinical intervention, though the prevalence rate of non-functional pituitary tumors remains high (5). Besides, pituitary adenomas can be grouped into microadenomas (diameter ≤ 10 mm) and macroadenomas (diameter > 10 mm) according to their size. For microadenomas, especially asymptomatic cases, keeping follow-up or receiving conservative treatment can be chosen. Furthermore, based on imaging findings, biological behavior, and pathological features, pituitary tumors can be divided into invasive and non-invasive. The invasive type is difficult to resect completely and the drug treatment had very limited effects.

As one kind of complex endocrine tumor, pituitary tumors seriously affect the quality of life and prognosis of patients because of their endocrinal nature and/or space-occupying effect. For PMPT, the invasive behavior made it more complex and diverse. In the present study, using the large cancer database with clinical information, the Surveillance, Epidemiology, and End Results (SEER) program proposed by the National Cancer Institute of the United States, we analyzed the incidence, demographics, treatments, long-term survival, and death causes of patients with primary malignant tumors located at the pituitary gland, including adenocarcinoma, invasive adenoma, germinoma, and chordoma. We aimed to first provide an overview of the malignant pituitary tumor in the past decades.

## Materials and methods

### Study patients

Using SEER*Stat software (version 8.3.8; https://seer.cancer.gov/seerstat/), the population-based registries from the National Cancer Institute's SEER database were retrieved and identified for this retrospective study, which covers approximately 30% of cancer patients in the United States with clinical information of incidence, demographic characteristics, survival, and death. Patients diagnosed with primary malignant tumors located in the pituitary gland were regarded as our study population. Due to the anonymity of the SEER database, the informed consent was waived and this study was approved to be exempted research by the Ethics Committee of West China Hospital. In this study, PMPT was defined as primary tumors with malignant behavior located in the pituitary gland, mainly including adenocarcinoma, invasive adenoma, germinoma, and chordoma.

### Incidence trend

To investigate the long-term incidence of pituitary tumors in the past decades, the SEER Research Data with nine Registries (1975–2017) was used to estimate the frequencies and incidence rates by SEER*Stat software, which had been updated in November 2019. Moreover, the annual percent change (APC) was calculated using a Join-point regression analysis program (version 4.8.0.1; https://surveillance.cancer.gov/joinpoint/) to describe the incidence trend over time. In the Join-point analysis, the optimal fitting piecewise continuous log-linear model was identified, and the permutation test was used for finding the minimal number of join points that fit the data.

### Demographics, treatments, and survival outcomes

In order to describe pituitary malignant tumor patients' characteristics, we retrieved the SEER 18 Registries Custom Data (with additional treatment fields; November 2018 Sub; 1975–2016). These cases with indefinite primary tumors or unknown follow-up were excluded. The demographic and treatment characteristics included age (pediatric: <18 and adult: ≥18 years), race (white, black, and others, involving American Indian/AK Native, Asian/Pacific Islander), year of diagnosis for the primary pituitary tumor, tumor extent staging (localized, regional, distant and unknown), tumor size, histology (adenocarcinoma, invasive adenoma, germinoma, chordoma, and others/unknown), marital status (married, single, widowed, divorced/separated, unknown), surgery (yes and no), radiotherapy (yes and no), radiation sequence (no, after surgery, before surgery and received but unknown sequence), and chemotherapy (yes and no).

Furthermore, distributions of patients' characteristics were compared between different populations of different ages (pediatric and adult), and univariate Cox regression analyses for the corresponding subgroups were performed. Those variables with a *P* value < 0.05 for all patients were further entered into multivariate Cox regression. Then, survival curves were plotted for variables with a significant difference by the Kaplan–Meier method and were compared using the log-rank test.

To evaluate the effects of treatments on patients with pituitary tumors, the long-term survival outcome was compared between treatments before and after the adjustment by propensity score matching (PSM) analyses (using the nearest neighbor matching algorithm; ratio: 1:1, caliper value = 0.05).

### Statistical analysis

The statistical analyses were performed using IBM SPSS 25.0 and R software (version 3.6.3; https://www.r-project.org). *P* value < 0.05 was considered statistically signiﬁcant. Distributions of clinical characteristics between pediatric and adult patients were compared by Pearson's Chi-square test (or Fisher's exact test if necessary). In the Cox regression analyses, hazards ratio with a 95% confidence interval (95%CI) was calculated.

## Results

### Incidence statistics

**Figure 1:**
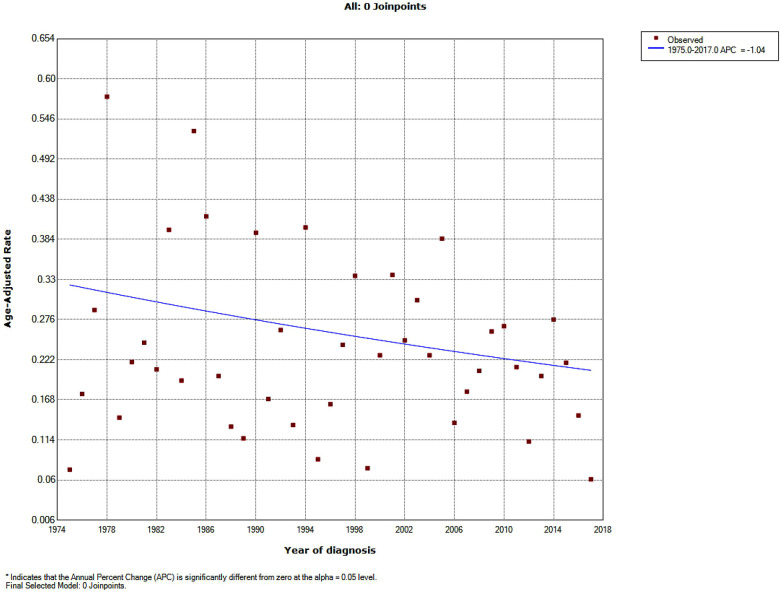
Incidence trend over years in patients with primary malignant pituitary tumor from the SEER research data with nine registries (1975–2017).

**Figure 2:**
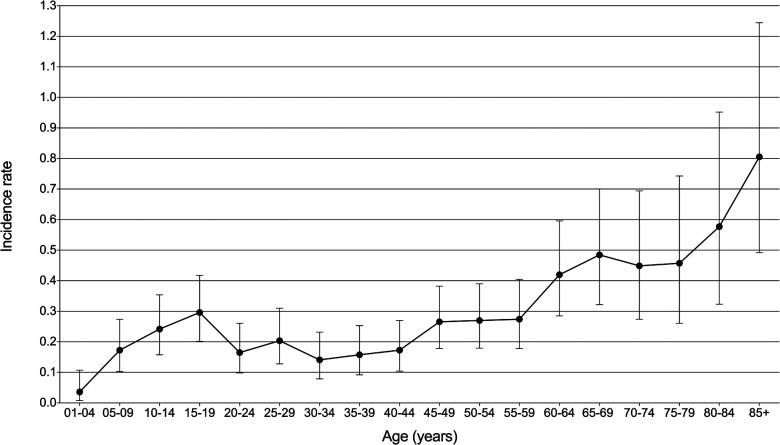
Incidence rate with 95% confidence interval at each age range in patients with primary malignant pituitary tumor from the SEER research data with nine registries (1975–2017).

A total of 251 patients diagnosed with PMPT between 1975 and 2017 from the 9 SEER registries of Research Data were identified with an average annual age-adjusted rate (AAR) of 0.233 (95%CI: 0.205–0.264) per 1,000,000 individuals. As shown in Figure 1, the age-adjusted incidence rate seemed to become lower over time from 1975 to 2017 without any join point, though there was no significance for the changing trend with an average APC of −1.04% (95%CI: −2.2%−0.1%, *P* = 0.10) per year. Besides, the crude incidence rates at each range of ages were also calculated and presented in Figure 2, from which it could be presumed that the rate increasingly changed over age among both pediatric and adult populations, but it suddenly declined at the adult age. The old patients with age ≥60 years demonstrated largely higher incidence rates than those with younger people.

### Demographic characteristics and survival analyses

From the SEER 18 Registries Custom Data (with additional treatment fields), a total of 501 patients diagnosed with PMPT between 1975 and 2016 finally met the inclusion criteria, involving 89 (17.8%) pediatric and 412 (82.2%) adult patients (Table 1). The univariate Cox regression analyses on all patients demonstrated that age (HR = 1.046, 95%CI: 1.038–1.053, *P* < 0.001; adult vs. pediatric, HR = 6.20, 95%CI: 3.28–11.70, *P* < 0.001), sex (female vs. male, HR = 0.69, 95%CI: 0.52–0.91, *P* = 0.008), tumor extent (regional vs. localized, HR = 2.26, 95%CI: 1.42–3.61, *P* = 0.001; distant vs. localized, HR = 5.36, 95%CI: 2.70–10.66, *P* < 0.001), histology (reference: adenocarcinoma; invasive adenoma: HR = 1.48, 95%CI: 0.54–4.03, *P* = 0.446; germinoma: HR = 0.33, 95%CI: 0.16–0.68, *P* = 0.003; chordoma: HR = 1.90, 95%CI: 1.03–3.50, *P* = 0.040; others/unknown: HR = 1.76, 95%CI: 1.06–2.91, *P* = 0.028), marital status (reference: married; single: HR = 0.38, 95%CI: 0.26–0.54, *P* < 0.001; widowed: HR = 2.47, 95%CI: 1.64–3.73, *P* < 0.001; divorced/separated: HR = 1.46, 95%CI: 0.90–2.39, *P* = 0.127), radiotherapy (yes vs. no, HR = 0.59, 95%CI: 0.44–0.79, *P* < 0.001), and chemotherapy (yes vs. no, HR = 0.50, 95%CI: 0.33–0.77, *P* = 0.002) were significantly associated with survival outcome. In addition, the cross-table analyses (as shown in Table 1) showed several characteristics were significantly differently distributed between pediatric and adult patients, including tumor extent (*P* = 0.003), histology (*P* < 0.001), marital status (*P* < 0.001), surgery (*P* = 0.017), radiotherapy (*P* < 0.001), radiation sequence (*P* = 0.007), and chemotherapy (*P* < 0.001).

**Table 1:** Univariate Cox regression analyses for overall survival in patients with primary malignant pituitary tumors.

Characteristic	Total (*N* = 501)			Pediatric (*N* = 89)			Adult (*N* = 412)			*P* value^a^
	*N* (%)	HR (95% CI)	*P* value	*N* (%)	HR (95% CI)	*P* value	N (%)	HR (95% CI)	*P* value	
Age, years	501 (100)	1.046 (1.038–1.053)	<0.001	89 (17.8)	—	—	412 (82.2)	—	—	—
Median (IQR)	46 (23-64)	—	—	12 (8-15)	—	—	53 (35–67)	—	—	—
Pediatric	81 (17.8)	Ref	—	—	—	—	—	—	—	
Adult	412 (82.2)	6.20 (3.28–11.70)	<0.001	—	—	—	—	—	—	
Sex										
Male	262 (52.3)	Ref	—	46 (51.7)	Ref	—	216 (52.4)	Ref	—	0.899
Female	239 (47.7)	0.69 (0.52–0.91)	0.008	43 (48.3)	0.46 (0.12–1.70)	0.242	196 (47.6)	0.68 (0.52–0.91)	0.008	
Race										
White	357 (71.3)	Ref	—	70 (78.7)	Ref	—	287 (69.7)	Ref	—	0.057
Black	82 (16.4)	1.33 (0.93–1.90)	0.119	7 (7.9)	<0.001 (0-∞)	0.989	75 (18.2)	1.15 (0.80–1.64)	0.459	
Others	62 (12.4)	0.41 (0.23–0.73)	0.003	12 (13.5)	2.42 (0.26–22.36)	0.437	50 (12.1)	0.34 (0.18–0.62)	<0.001	
Diagnosis year										
1975–1990	85 (17)	Ref	—	12 (13.5)	Ref	—	73 (17.7)	Ref	—	0.556
1991–2000	76 (15.2)	1.24 (0.82–1.88)	0.315	17 (19.1)	3.39 (0.23–50.47)	0.376	59 (14.3)	1.45 (0.95–2.22)	0.087	
2001–2005	114 (22.8)	0.93 0.62–1.41)	0.731	18 (20.2)	5.34 (0.26–109.27)	0.277	96 (23.3)	0.91 (0.60–1.38)	0.659	
2006–2010	110 (22)	1.03 (0.66–1.60)	0.894	18 (20.2)	0 (0-∞)	0.974	92 (22.3)	1.08 (0.69–1.68)	0.739	
2011–2016	116 (23.2)	0.85 (0.49–1.45)	0.543	24 (27)	7.97 (0.23–272.79)	0.25	92 (22.3)	0.88 (0.51–1.52)	0.642	
Tumor extent										
Localized	87 (17.4)	Ref	—	27 (30.3)	Ref	—	60 (14.6)	Ref	—	0.003
Regional	59 (11.8)	2.26 (1.42–3.61)	0.001	10 (11.2)	1.38 (0.32–5.89)	0.666	49 (11.9)	2.09 (1.26–3.44)	0.004	
Distant	14 (2.8)	5.36 (2.70–10.66)	<0.001	0	—	—	14 (3.4)	3.79 (1.89–7.63)	<0.001	
Unknown	341 (68.1)	1.99 (1.35–2.93)	0.001	52 (58.4)	1.17 (0.16–8.68)	0.879	289 (70.1)	1.67 (1.11–2.52)	0.013	
Tumor size, cm										
≤1.0	15 (3)	Ref	—	5 (5.6)	Ref	—	10 (2.4)	Ref	—	0.251
1.1–2.0	41 (8.2)	1.64 (0.35–7.75)	0.529	10 (11.2)	1.04 (0-∞)	0.998	31 (7.5)	1.67 (0.35–7.86)	0.518	
2.1–3.0	51 (10.2)	3.08 (0.71–13.35)	0.132	6 (6.7)	1.07 (0-∞)	0.997	45 (10.9)	2.76 (0.64–11.96)	0.174	
>3.0	52 (10.4)	2.37 (0.54–10.41)	0.255	10 (11.2)	1.05 (0-∞)	0.998	42 (10.2)	2.19 (0.50–9.66)	0.299	
Unknown	342 (68.3)	2.97 (0.74–12.00)	0.127	58 (65.2)	36.68 (0-∞)	0.806	284 (68.9)	2.69 (0.67–10.87)	0.165	
Histology										
Adenocarcinoma	39 (7.8)	Ref	—	1 (1.1)	Ref	—	38 (9.2)	Ref	—	<0.001
Invasive adenoma	15 (3)	1.48 (0.54–4.03)	0.446	2 (2.2)	0.98 (0-∞)	1	13 (3.2)	1.58 (0.58–4.32)	0.37	
Germinoma	103 (20.6)	0.33 (0.16–0.68)	0.003	60 (67.4)	946.42 (0-∞)	0.961	43 (10.4)	0.52 (0.22–1.20)	0.123	
Chordoma	45 (9)	1.90 (1.03–3.50)	0.04	1 (1.1)	—	—	44 (10.7)	1.84 (1.00–3.38)	0.052	
Others/unknown	299 (59.7)	1.76 (1.06–2.91)	0.028	25 (28.1)	1650.22 (0-∞)	0.958	274 (66.5)	1.95 (1.17–3.23)	0.01	
Marital status										
Married	202 (40.3)	Ref	—	0	—	—	202 (49)	Ref	—	<0.001
Single (never married)	197 (39.3)	0.38 (0.26–0.54)	<0.001	89 (100%)	—	—	108 (26.2)	0.63 (0.42–0.93)	0.02	
Widowed	35 (7)	2.47 (1.64–3.73)	<0.001	0	—	—	35 (8.5)	2.42 (1.61–3.65)	<0.001	
Divorced/separated	32 (6.4)	1.46 (0.90–2.39)	0.127	0	—	—	32 (7.8)	1.45 (0.89–2.36)	0.141	
Unknown	35 (7)	0.95 (0.56–1.60)	0.834	0	—	—	35 (8.5)	0.93 (0.55–1.57)	0.78	
Surgery										
No	150 (29.9)	Ref	—	36 (40.4)	Ref	—	114 (27.7)	Ref	—	0.017
Yes	351 (70.1)	0.86 (0.63–1.18)	0.35	53 (59.6)	0.80 (0.13–4.84)	0.806	298 (72.3)	0.75 (0.55–1.03)	0.076	
Radiotherapy										
No	299 (59.7)	Ref	—	23 (25.8)	Ref	—	276 (67.0)	Ref	—	<0.001
Yes	202 (40.3)	0.59 (0.44–0.79)	<0.001	66 (74.2)	0.90 (0.18–4.46)	0.894	136 (33.0)	0.76 (0.57–1.03)	0.075	
Radiation sequence										
No radiation	344 (68.7)	Ref	—	48 (53.9)	Ref	—	296 (71.8)	Ref	—	0.007
After surgery	135 (26.9)	0.64 (0.46–0.90)	0.899	35 (39.3)	2.07 (0.38–11.33)	0.401	100 (24.3)	0.69 (0.49–0.98)	0.036	
Before surgery	3 (0.6)	0.32 (0.05–2.31)	2.313	1 (1.1)	0 (0-%)	0.99	2 (0.5)	0.36 (0.05–2.61)	0.313	
Unknown	19 (3.8)	0.90 (0.51–1.57)	1.574	5 (5.6)	1.95 (0.24–16.23)	0.536	14 (3.4)	1.05 (0.58–1.91)	0.875	
Chemotherapy										
No	395 (78.8)	Ref	—	30 (33.7)	Ref	—	365 (88.6)	Ref	—	<0.001
Yes	106 (21.2)	0.50 (0.33–0.77)	0.002	59 (66.3)	1.03 (0.24–4.48)	0.964	47 (11.4)	1.15 (0.72–1.82)	0.57	

^a^ Chi-square test.

HR, hazards ratio; IQR, interquartile range.

Then, the significant variables in the univariate Cox regression analyses further entered into multivariate analysis, which demonstrated that age (adult vs. pediatric, HR = 3.18, 95%CI: 1.49–6.82, *P* = 0.003), sex (female vs. male, HR = 0.56, 95%CI: 0.41–0.74, *P* < 0.001), tumor extent (regional vs. localized, HR = 1.82, 95%CI: 1.12–2.95, *P* = 0.015; distant vs. localized, HR = 4.01, 95%CI: 1.96–8.21, *P* < 0.001) and marital status (reference: married; single: HR = 0.62, 95%CI: 0.41–0.93, *P* < 0.001; widowed: HR = 2.47, 95%CI: 1.62–3.75, *P* < 0.001; divorced/separated: HR = 1.72, 95%CI: 1.05–2.84, *P* = 0.033) were independent prognostic factors for pituitary tumor (Table 2).

**Table 2:** Multivariate Cox regression analysis for overall survival in patients with primary malignant pituitary tumors.

Characteristic	HR	95%CI	*P* value
Age			
Pediatric	Ref	—	—
Adult	3.18	1.49–6.82	0.003
Sex			
Male	Ref	—	—
Female	0.56	0.41–0.74	<0.001
Tumor extent			
Localized	Ref	—	—
Regional	1.82	1.12–2.95	0.015
Distant	4.01	1.96–8.21	<0.001
Unknown	1.35	0.90–2.03	0.153
Histology			
Adenocarcinoma	Ref	—	—
Invasive adenoma	2.12	0.75–5.94	0.155
Germinoma	0.81	0.36–1.80	0.596
Chordoma	1.62	0.86–3.06	0.137
Others/unknown	2.03	1.20–3.43	0.008
Marital status			
Married	Ref	—	—
Single (never married)	0.62	0.41–0.93	0.022
Widowed	2.47	1.62–3.75	<0.001
Divorced/separated	1.72	1.05–2.84	0.033
Unknown	0.95	0.56–1.63	0.861
Radiotherapy			
No	Ref	—	—
Yes	0.96	0.70–1.30	0.785
Chemotherapy			
No	Ref	—	—
Yes	1.59	0.97–2.60	0.064

**Figure 3:**
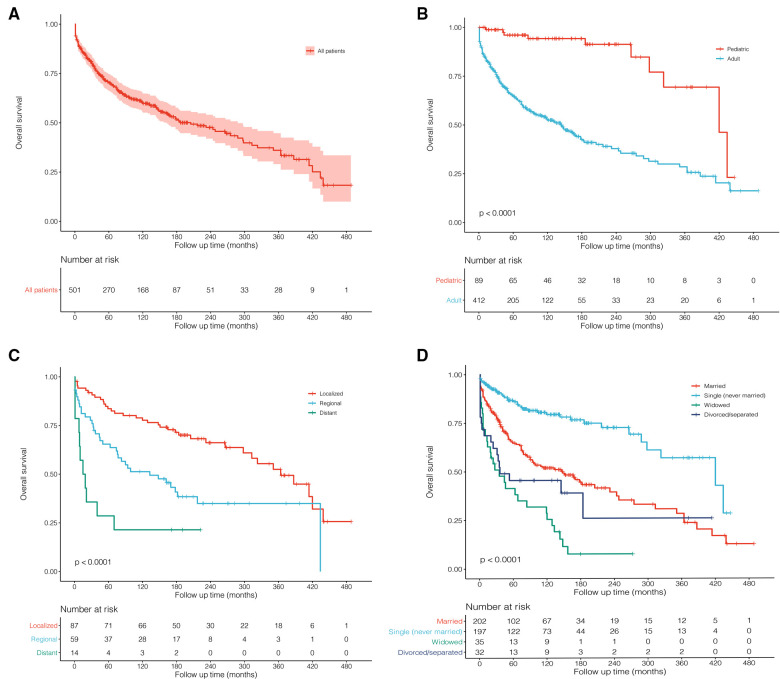
Kaplan–Meier survival curves and log-rank tests for possible prognostic factors. (**A**) All 501 patients were diagnosed with primary malignant pituitary tumors between 1975 and 2016 from the SEER 18 Registries Custom Data. (**B**) Age: pediatric and adult. (**C**) Tumor extent: localized, regional, and distant. (**D**) Marital status: married, single (never married), widowed, and divorced/separated.

For all enrolled patients, the mean follow-up was 102 months with a median survival time (MST) of 205 months (Figure 1). According to the results of multivariate Cox regression, the significant prognostic factors (age, tumor extent, and marital status) were used for plotting Kaplan–Meier survival curves. The pediatric patients (MST: 420 months) showed significantly better long-term survival than adults (MST: 145 months; Figure 3B, *P* < 0.001). Obviously, localized stage patients also demonstrated better survival than the regional stage, followed by the distant stage with the worst survival (Figure 3C, *P* < 0.001). As for marital status, the never-married patients had better survival outcomes than others, and the widowed people performed worst (Figure 3D, *P* < 0.001).

### Treatments

**Figure 4:**
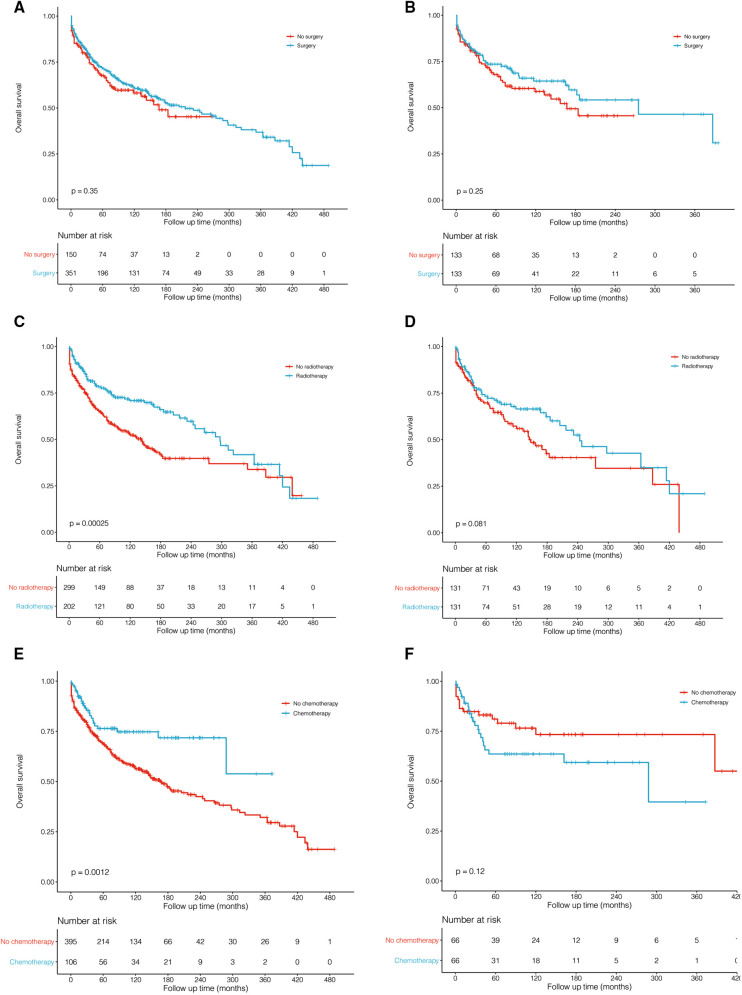
Kaplan–Meier survival curves and log-rank tests for treatments before (**A,C,E**) and after (**B,D,F**) propensity score matching (PSM). (**A,B**). Surgery vs. no surgery. (**C,D**) Radiotherapy vs. no radiotherapy. (**E,F**) Chemotherapy vs. no chemotherapy.

Table 3 describes treatment patterns for patients with PMPT. The most common therapy for them was surgery plus radiotherapy (MST: 297 months) and only surgery (MST: 143 months). To evaluate the prognostic effects of these treatments, PSM analyses were performed to balance the remaining characteristics. Surgical resection did not benefit the survival outcome of pituitary tumors (Figure 4A, *P* = 0.350; Figure 4B, *P* = 0.250). Before matching, patients undergoing surgery, radiotherapy, and chemotherapy showed better survival than those who did not (Figure 4C, *P* < 0.001; Figure 4E, *P* < 0.001). However, the significant differences faded away after PSM (Figure 4D, *P* = 0.081; Figure 4F, *P* = 0.120).

**Table 3:** Number and median survival time (MST) of patients with primary malignant pituitary tumors in different treatment groups.

Treatment	Total (*N* = 501)		Pediatric (*N* = 89)		Adult (*N* = 412)	
	*N* (%)	MST	*N* (%)	MST	*N* (%)	MST
All patients	501	205	89	420	412	145
None	95	119	5 (5.3)	—	90 (94.7)	84
Only surgery	177	143	7 (4.0)	—	170 (96.0)	129
Only radiotherapy	16	167	2 (12.5)	—	14 (87.5)	167
Only chemotherapy	10	—	6 (60.0)	—	4 (40.0)	—
Radiotherapy + chemotherapy	29	—	23 (79.3)	—	6 (20.7)	—
Surgery + radiotherapy	107	297	16 (15.0)	—	91 (85.0)	232
Surgery + chemotherapy	17	—	5 (29.4)	—	12 (70.6)	—
Surgery + radiotherapy + chemotherapy	50	288	25 (50.0)	—	25 (50.0)	162

### Death causes

**Figure 5:**
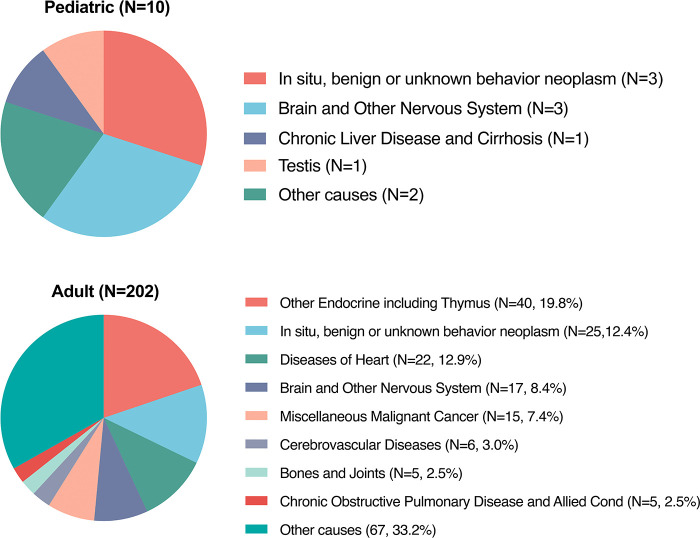
Causes of death among pediatric and adult patients with primary malignant pituitary tumor from the SEER 18 registries custom data.

Until the last update of follow-up in November 2018, there was a sum of 212 (42.3%, 212/501) PMPT patients who died, which involved 10 (11.2%, 10/89) pediatric cases and 202 (49.0%, 202/412) adults. Figure 5 shows death causes of pediatric and adult patients. Ten pediatric deaths included benign or unknown behavior neoplasm (*in situ*; *N* = 3), brain and other nervous systems (*N* = 3), chronic liver disease and cirrhosis (*N* = 1), testis disease (*N* = 1), and other unknown causes (*N* = 2). Among adults, five of the most common causes were other endocrine diseases (*N* = 40, 19.8%), benign or unknown behavior neoplasm (*in situ*; *N* = 25, 12.4%), heart diseases (*N* = 22, 12.9%), brain and other nervous systems (*N* = 17, 8.4%); miscellaneous malignant cancer (*N* = 15, 7.4%).

## Discussion

A pituitary tumor is a kind of common intracranial tumor that has been poorly studied in previous pieces of literature. It was currently considered that pituitary tumors, especially adenoma, originated from the abnormal differentiation of anterior pituitary cells or craniofacial epithelial cells. At present, the treatment for pituitary adenomas includes drug therapy, radiotherapy, and surgery, among which surgery is the most effective and thorough method (6). Clinically, pituitary adenomas can be divided into functional and non-functional types. The active endocrine state is closely related to the functioning tumors, which can lead to a series of symptoms, such as acromegaly caused by high levels of growth hormone and insulin growth factor one, amenorrhea or sexual dysfunction caused by hyperprolactinemia, Cushing's disease caused by hypercorticosteremia, etc (7). The non-functional tumors do not cause hypersecretory symptoms and signs but have more obvious space-occupying effects, which can show several manifestations including headache, visual field defect, visual acuity, hypophysis, etc (8, 9). However, few studies focused on the rare malignant tumor at the pituitary gland, mainly including adenocarcinoma, invasive adenoma, germinoma, and chordoma. We aimed in this retrospective cohort study to provide an overview of the incidence, demographics, treatments, and long-term survival of PMPT patients. Moreover, causes of death were also first analyzed among these patients.

The incidence of pituitary tumors varied in different reports (1, 10, 11). The average prevalence rate of pituitary adenomas in autopsy data was estimated to be about 10.7% (11). PMPT occurred more rarely. According to the SEER data, the AAR of PCL between 1975 and 2017 was 0.233 per 1,000,000 population. The rate increasingly changed over age among both pediatric and adult populations. These results were consistent with the previous report of the Central Brain Tumor Registry of the United States (CBTRUS) between 2004 and 2009 (12), which demonstrated that malignant pituitary tumors occurred most commonly among 65–74 years old and were most rare among those 15–24 years old (Figure 2). However, the incidence rate of malignant pituitary tumors seemed to become lower over time from 1975 to 2017 without any join point, the APC was −1.04% per year (Figure 1). This study first described the changing trend of incidence in patients with a malignant pituitary tumor in the United States, but we could not estimate the rates of pituitary adenoma for comparisons due to the limitations of the SEER database.

Our analysis showed that age (adult vs. pediatric, HR = 3.18, 95%CI: 1.49–6.82, *P* = 0.003; Table 2) was also an independent prognostic factor for pituitary tumors. The older age (HR = 1.046, 95%CI: 1.038–1.053, *P* < 0.001, Table 1) and male gender (female vs. male, HR = 0.56, 95%CI: 0.41–0.74, *P* < 0.001; Table 2) were significantly associated with poorer prognosis. Additionally, among all patients, histological types seemed not to affect overall survival outcomes. However, marital status was surprisingly identified as a significant prognostic factor. Compared with married patients, the single status cases had a better prognosis (HR = 0.62, 95%CI: 0.41–0.93, *P* = 0.022), but the widowed (HR = 2.47, 95%CI: 1.62–3.75, *P* < 0.001) and the divorced/separated (HR = 1.72, 95%CI: 1.05–2.84, *P* = 0.033) indicated a higher risk of poor prognosis, which was not reported before. The results may be attributed to the confounding effect of different ages, for most patients with single status (never married) were at a younger age. The previous research reported an increase in mortality among widowed and never-married cancer patients (13), partly consistent with our study. These results may present the critical effects of marital status in the management and survival outcomes for cancer patients.

Patients with pituitary tumors showed great survival outcomes. In our study, a total of 501 patients were identified with an MST of 205 months (Table 3). The pediatric (MST = 420 months) demonstrated significant overall survival in adults (MST = 145 months; *P* < 0.001; Figure 3). Pituitary tumors are commonly and easily treated by surgery or other medical treatments in most cases. Radiation therapy is usually considered when initial treatment fails or disease recurs. It was previously reported that pituitary surgery was the most effective treatment for most endocrinal pituitary adenomas, non-functioning pituitary adenomas causing mass effect, and pituitary cancer (14). In this cohort that we studied, the most commonly used therapy for them was surgery plus radiotherapy (MST: 297 months; Table 3) and only surgery (MST: 143 months). However, surgery may not be an effective treatment for our cohort (Figure 4; before matching: *P* = 0.35, after matching: *P* = 0.25), which only included pituitary tumors with malignant behavior. Furthermore, radiotherapy and chemotherapy may also not significantly affect overall survival after matching among malignant pituitary tumors (Figure 4). Considering the limited number of patients, future studies are required to focus on systematical therapy for malignant pituitary tumors.

The present study also first reported the death causes among malignant primary tumors (Figure 5). Among the 212 deaths, most were adults (202/212, 95.3%). Among adults, the most common causes were from other endocrine organs (*N* = 40, 19.8%), followed by unknown neoplasm (*N* = 25, 12.4%), heart diseases (*N* = 22, 12.9%), and brain and other nervous systems (*N* = 17, 8.4%), which indicated that few patients died of pituitary tumors.

Several limitations of this study should be considered. First, the retrospective cohort was retrieved from the SEER database, and some natural data bias cannot be avoided totally. Second, the database did not include some important characteristics such as imaging features, detailed information on chemotherapy and radiotherapy, etc. Third, the SEER database only included tumors with malignant behavior, while most pituitary adenomas were actually benign. Thus, we could not compare characteristics and survival between malignant and benign pituitary tumors.

## Conclusion

In conclusion, the AAR for PMPT from 1975 to 2017 was 0.233 (95%CI: 0.205–0.264) per million population. The Join-point analysis indicated an insignificantly decrease in incidence rates over years. Older age, tumor extent, and marital status were independent prognostic factors for PMPT. The PSM analyses demonstrated no significant survival benefits from surgery, radiotherapy, and chemotherapy among PMPT patients. More future studies should be carried on to focus on therapy.

## Data Availability

The original contributions presented in the study are included in the article/Supplementary Material, further inquiries can be directed to the corresponding author/s.
